# Clinical and parasitological impact of short-term treatment using miltefosine and allopurinol monotherapy or combination therapy in canine visceral leishmaniasis

**DOI:** 10.1590/S1984-29612022040

**Published:** 2022-08-01

**Authors:** Eveline da Cruz Boa Sorte Ayres, Álvaro Felipe de Lima Ruy Dias, Bruna Ribeiro Gomes Monteiro, Sarah Szimanski Pazzini, Mateus Elias Chagas Barbosa, Eveliny Barroso da Silva, Luis Felipe da Cruz Macedo, Valéria Régia Franco Sousa, Valéria Dutra, Luciano Nakazato, Arleana do Bom Parto Ferreira de Almeida

**Affiliations:** 1 Laboratório de Leishmanioses, Hospital Veterinário, Faculdade de Medicina Veterinária, Universidade Federal de Mato Grosso – UFMT, Cuiabá, MT, Brasil; 2 Laboratório de Estatística. Departamento de Estatística, Instituto de Ciências Exatas e da Terra, Universidade Federal de Mato Grosso – UFMT, Cuiabá, MT, Brasil; 3 Laboratório de Farmacologia, Faculdade de Medicina, Universidade Federal de Mato Grosso – UFMT, Cuiabá, MT, Brasil; 4 Laboratório de Biologia Molecular, Hospital Veterinário, Faculdade de Medicina Veterinária, Universidade Federal de Mato Grosso – UFMT, Cuiabá, MT, Brasil

**Keywords:** Miltefosine, Allopurinol, Leishmania infantum, qPCR, skin, Miltefosina, Alopurinol, Leishmania infantum, qPCR, pele

## Abstract

Canine visceral leishmaniasis is an endemic zoonosis in Brazil. Dogs are the main hosts in urban environments. The treatment has gained popularity since the Brazilian government authorized miltefosine for canine treatment. The aim of this study was to investigate the clinical and parasitological impact of short-term treatment with miltefosine and allopurinol, alone and in combination. We evaluated the ability of pharmacotherapy to reduce clinical signs of disease, antibody levels using the indirect fluorescence antibody test (IFAT) and skin parasite load via qPCR after 28 days of treatment. The therapeutic protocols promoted a significant decline in clinical signs and in the skin parasite load in dogs (p < 0.01). We observed a moderate correlation between the skin parasite load and the clinical score in all three treatment groups (r > 0.5) Antibody levels did not decrease in this short period. It was concluded that the treatment with allopurinol reduced the number of parasites in the skin of dogs with visceral leishmaniasis in the short term. However, its efficiency is potentiated when associated with miltefosine.

## Resumo

A leishmaniose visceral canina é uma zoonose endêmica no Brasil. Os cães são os principais hospedeiros em ambientes urbanos. O tratamento ganhou popularidade desde que o governo brasileiro autorizou a miltefosina para tratamento canino. O objetivo deste estudo foi investigar o impacto clínico e parasitológico do tratamento a curto prazo com miltefosina e alopurinol, isoladamente e/ou em combinação. Foi avaliada a capacidade da farmacoterapia em reduzir os sinais clínicos da doença e também os níveis de anticorpos, usando-se o teste de anticorpos de fluorescência indireta (RIFI) e a carga parasitária na pele, via qPCR, após 28 dias de tratamento. Os protocolos terapêuticos promoveram declínio significativo dos sinais clínicos e da carga parasitária na pele dos cães (p < 0,01). Foi observada uma correlação moderada entre a carga parasitária da pele e o escore clínico em todos os três grupos de tratamento (r > 0,5). Já os níveis de anticorpos não diminuíram nesse curto período. Concluiu-se que o tratamento com alopurinol, em curto prazo, reduziu a quantidade de parasitos na pele dos cães com leishmaniose visceral. No entanto, sua eficiência é potencializada quando associada a miltefosina.

## Introduction

Visceral leishmaniasis (VL) is a global public health problem with the potential for outbreaks and mortality. While India, Bangladesh, and Nepal have progressively reduced the incidence of this disease in humans in the last decade, Brazil has reported an increase in the number of cases (Brasil, 2019; Dahl et al., 2021; WHO, 2021). In Brazil, VL is caused by the protozoan, *Leishmania infantum*, and the main vector is the sandfly, *Lutzomyia longipalpis* (Borges et al., 2021; Sevá et al., 2020; WHO, 2021). In the past, VL predominantly existed in rural environments. However, the disease has adapted to the urban environment due to environmental changes, such as deforestation and urbanization (Santos et al., 2021). Due to the adaptation of the sand fly to the domestic environment, dogs have become the main source of infection for humans in urban areas (Marcondes & Day, 2019; Scorza et al., 2021).

Dogs can present subclinical infections, acquire protective immunity, or manifest severe classic clinical signs (Marcondes & Day, 2019). The most common clinical signs include skin lesions such as ulcers, alopecia, and scaling, lymphadenomegaly, anorexia, muscle atrophy, lethargy, splenomegaly, eye lesions, onychogryphosis, lameness, vomiting, and diarrhea (Ciaramella et al., 1997; Solano-Gallego et al., 2009). Even when clinical signs are absent, infected dogs can exhibit severe cutaneous parasitism with infectious potential (Chagas et al., 2021; Scorza et al., 2021).

The diagnostic tests recommended by the Brazilian Ministry of Health are Dual-path Platform chromatographic immunoassay (DPP® CVL rapid test) and ELISA. However, these tests have shown low sensitivity to estimate prevalence in canines (Borges et al., 2021). PCR detection of *Leishmania* DNA has accelerated the diagnosis and has been used to identify species and their geographic distribution, thereby improving disease surveillance (Hong et al., 2020). Quantitative PCR (qPCR) is the gold standard test for quantifying the parasite load in different tissues of infected dogs with or without clinical manifestations (Manna et al., 2008). It can be used to diagnose and monitor infection during treatment (Francino et al., 2006; Chagas et al., 2021).

Currently, miltefosine is the only drug authorized for canine treatment in Brazil and is clinically effective (Nogueira et al., 2019). In several studies, the efficacy of miltefosine has been associated with the use of allopurinol which is administered mainly to prevent the disease recurrence (Pennisi et al., 2005; Yasur-Landau et al., 2017; Santos et al., 2020), although allopurinol effectively reduces the parasite load used alone (Nascimento et al., 2020). However, in Brazil, allopurinol is not yet authorized as a veterinary drug for canine VL (CVL) therapy and has been used only in research (Dias et al., 2020). Dogs responsive to treatment exhibit clinical improvement and a reduced parasite load on the skin, consequently posing a decreased risk of transmission (Santos et al., 2019).

In Europe, miltefosine has been part of the canine therapeutic protocol for almost 20 years (Sindermann & Engel, 2006; Woerly et al., 2009; Nogueira et al., 2019). In Brazil, its use is recent and has limitations, such as a high cost and lack of incentive from the state since canine treatment is not classified as an official preventive measure for human VL (Brasil, 2014). Additionally, official measures for monitoring dogs undergoing treatment do not exist (Nunes et al., 2018). Therefore, this study aimed to evaluate the effectiveness of miltefosine and allopurinol, used alone and in combination, by observing the impact of treatment on the clinical signs, skin parasite load, and antibody titers in naturally infected dogs using qPCR and the indirect fluorescence antibody test (IFAT).

## Materials and methods

### Study location

This clinical study was conducted from June 2017 to October 2019 at the Veterinary Hospital of the Federal University of Mato Grosso (HOVET-UFMT), Cuiabá campus, Brazil (geographic coordinates 15º35'56” S/56º06'01” W), which is in an area endemic for human and canine VL. Individuals interested in treating their dogs signed an informed consent and commitment form authorizing the collection of biological materials. The study was approved by the Ethics Committee on the Use of Animals (CEUA/UFMT) and registered under protocol number 23108.025481/2019-81.

### Selection of animals

For this study, 45 dogs naturally infected with *L. infantum*, diagnosed by serological, parasitological, and/or molecular tests, were selected. These dogs belonged to different breeds and both sexes. Dogs that were over six months old, were neither pregnant nor lactating, and had no history of vaccination or previous use of drugs with leishmanicidal and/or leishmaniostatic action were included in this study.

### Treatment protocols

The therapeutic protocols were prepared in accordance with the ethical principles for animal experimentation of the Brazilian College of Animal Experimentation (COBEA). The dogs were evenly divided into three groups (n = 15/group) and received the following treatment protocols: Group 1 (G1): 15 dogs treated with miltefosine (Milteforan™, Virbac), 2 mg/kg, orally, once a day for 28 consecutive days (Nogueira et al., 2019); Group 2 (G2): 15 dogs treated with allopurinol, 20 mg/kg, orally, twice a day for 28 consecutive days (Dias et al., 2020); Group 3 (G3): 15 dogs treated with miltefosine in combination with allopurinol, at the exact dosages administered in Groups 1 and 2.

### Clinical signs

The dogs underwent clinical evaluation and sample collection one day before starting the treatment (D0) and one day after the last day of treatment (D29). They were categorized into asymptomatic and symptomatic groups according to the classification described by Solano-Gallego et al. (2009). Clinical signs were also scored on a severity scale from 0 to 3, in which 0 represents the absence of the evaluated symptom and 3 represents a severe symptom, with a maximum total score of 86 (Proverbio et al., 2014).

### Collection of biological materials

Dogs were sedated with ketamine hydrochloride and acepromazine at an intramuscular (IM) dose of 10 mg/kg and 0.2 mg/kg, respectively. Then, 5 mL of whole blood was collected by cephalic or external jugular venipuncture with immediate serum extraction for serological analysis by indirect immunofluorescence. With the dog in lateral recumbency, a 3 mm fragment of the intact skin was obtained by biopsy of the scapular region after shaving, asepsis, and local anesthesia with 2% lidocaine and stored at −80 °C for subsequent DNA extraction and qPCR analysis of the parasite load.

### Indirect Immunofluorescence (IFAT)

Anti-*Leishmania* IgG antibody titers were determined at D0 and D29 using a commercial LVC kit Bio-Manguinhos®, Fiocruz (Brazil) according to the manufacturer's recommendations. Negative and positive controls were included for each slide. Samples were considered positive when a uniform fluorescence reaction was observed at a serum dilution ≥1:80 (Brasil, 2014).

### Quantitative real-time PCR (qPCR)

DNA extraction from the skin samples was performed using the phenol/chloroform method (Sambrook & Russel, 2001) and subsequently quantified using the NanoDrop™ 2000/2000c Spectrophotometer (Thermo Scientific). To verify DNA integrity and the absence of inhibitors, DNA samples were tested for the endogenous canine β-globin gene (5'-CAA CTT CAT CCA CGT TCA CC-3' and 5'-ACA CAA CTG TGT TCA CTA GC -3'), which was used as an internal amplification control (Quaresma et al., 2009).

qPCR was performed in triplicate using the StepOne™ Real-Time PCR System Sequence Detection System (Applied Biosystems) with primers RV1–5'-CTT TTC TGG TCC GGG TAG G-3' and RV2–5'-CCA CCT GGC TAT TTT ACA CCA-3', which were used to amplify a 145 bp sequence of *L. infantum*-specific kinetoplast DNA (kDNA) (Lachaud et al., 2002). Reactions were prepared in a final volume of 25 μL containing SYBR Green Master Mix, 0.3 μM of each primer, and 2 μL of target DNA. The amplification conditions included an initial incubation step at 94 °C for 10 min, followed by 40 cycles of amplification at 94 °C for 15 s and 60 °C for 60 s. A standard curve was established for each assay using known amounts of TOPO PCR 2.1 plasmid (Invitrogen Corp.) containing the *L. infantum* kDNA gene. Serial dilutions (10×) containing 2 × 10^4^ 2 × 10^8^ copies of the recombinant plasmid were performed and used to establish the standard curve. A negative control containing DNA-free water was used in all reactions.

### Efficacy criteria for therapeutic protocols

Dogs were monitored during the 28 days of treatment, with clinical and laboratory assessments performed before (D0) and after (D29) completion of treatment. The efficacy was determined by examining the ability of the therapeutic protocols to reduce the clinical score, IFAT antibody titers, and qPCR-derived *L. infantum* parasite load in the skin of dogs.

### Statistical analysis

The Shapiro–Wilk test was used to assess the normality of data. For variables with a normal distribution, the paired t-test was used, and for non-parametric samples, the Wilcoxon signed-rank test was used. Statistical significance was set at P < 0.05. Pearson's correlation (r) was used to measure the relationship between the parasite load and clinical signs and the parasite load and antibody titers. The degree of correlation between the variables was indicated by values between −1 and 1. The tests were performed using R statistical software (R Core Team, 2019).

## Results

### Clinical signs

All 45 dogs participating in this study showed clinical signs of CVL on D0. The clinical alterations presented on D0 and D29 are shown in Table 1. Skin diseases were the most frequently observed clinical alterations present in 95.5% (43/45) of the dogs; in particular, skin ulcers, onychogryphosis, and alopecia were the most common alterations. Lymphadenomegaly (80%), anorexia (75%), apathy (64.4%), and weight loss (64.4%) were other frequently observed clinical signs. Ophthalmic alterations were present in 55.5% (25/45) of the dogs, the most frequent types being conjunctivitis, uveitis, and blepharitis.

**Table 1:** Clinical changes found in 45 dogs evaluated before and after 28 days of treatments.

Clinical Signs	Miltefosine		Allopurinol		Miltefosine + Allopurinol	
	**D0** (n / %)	**D29** (n / %)	**D0** (n / %)	**D29** (n / %)	**D0** (n / %)	**D29** (n / %)
Anorexia	13 / 86.7	8 / 53.3	11 / 73.3	3 / 20	10 / 66.7	5 / 33.3
Apathy	11 / 73.3	4 / 27	7 / 46.7	2 / 13.3	11 /73.3	2 / 13.3
Weigh loss	10 / 66.7	6 / 40	11 / 73.3	6 / 40	8 / 53.3	5 / 33.3
Lymph adenomegaly	12 / 80	10 / 66.7	11 / 73.3	6 / 40	13 / 86.7	10 / 66.7
Muscle atrophy	4 / 27	2 / 13.3	3 / 20	1 / 6.7	1 / 6.7	11 / 6.7
Arthropathy	3 / 20	1 / 6.7	7 / 46.7	3 / 20	6 / 40	5 / 33.3
Digestive Disorders	3 / 20	3 / 20	1 / 6.7	0 / 0	3 / 20	0 / 0
Splenomegaly	1 / 6.7	0 / 0	5 / 33.3	3 / 20	6 / 40	2 / 13.3
Polyuria/polydipsia	3 / 20	3 / 20	5 / 33.3	3 / 20	3 / 20	1 / 6.7
Epistaxis	3 / 20	1 / 6.7	2 / 13.3	0 / 0	1 / 6.7	0 / 0
Pale mucous membranes	5 / 33.3	5 / 33.3	6 / 40	3 / 20	7 / 46.7	2 / 13.3
Dermatological changes	14 / 93.4	13 / 86.7	15 / 100	13 / 86.7	14 / 93.4	14 / 93.4
Ophthalmic changes	8 / 53.3	7 / 46.7	5 / 33.3	4 / 27	12 / 80	7 / 46.7

The mean clinical score on D29 was significantly lower than that on D0 in all treated dogs, regardless of the protocol adopted (p < 0.01) (Table 2). Although there was no difference in mean clinical score between the groups, the percentage of reduced clinical scores were 36.9% in G1, 58.4% in G2, and 52.6% in G3. In G2, the allopurinol monotherapy group, one dog showed complete clinical remission and was classified as asymptomatic.

**Table 2:** Mean and Standard Deviation found in the clinical score and antibody titers by IFAT in dogs treated with the protocols miltefosine (G1), allopurinol (G2) and miltefosine combined with allopurinol (G3), before and after the therapies (n = 15/group).

	**Miltefosine (n = 15)**		
	**D0**	**D29**	**P-value**
**Clinical Signs**	12.5 ± 6.0	7.8 ± 5,5	< 0.01
**IFAT**	376.0 ± 267.8	405.0 ± 246.5	0.76
	**Allopurinol (n = 15)**		
	**D0**	**D29**	**P-value**
**Clinical Signs**	14.2 ± 8.4	5.7 ± 5.3	< 0.01
**IFAT**	285.3 ± 203.3	381.3 ± 209.9	0.99
	**Miltefosine + Allopurinol (n = 15)**		
	**D0**	**D29**	**P-value**
**Clinical Signs**	14.5 ± 7.4	6.8 ± 4.8	< 0.01
**IFAT**	354.6 ± 257.8	429.3 ± 191.5	0.82

The values ​​described are the Mean ± SD (95% CI) of the clinical scores and antibody titers by IFAT on D0 and D29 of the groups treated with three different protocols. P values ​​were obtained with the paired t-test.

### IFAT antibody titer

The antibody titer was ≥1:80 in 93.3% (42/45) of the animals before treatment. Samples from three dogs, one from each group, exhibited fluorescence only at a 1:40 dilution; therefore, these dogs were considered seronegative on D0. However, two of these dogs, one from G1 and one from G2, seroconverted on D29. None of the therapeutic protocols reduced anti-*Leishmania* IgG antibody titers after 28 days of treatment (p > 0.05) (Table 3). G2 presented a less significant antibody titer (p = 0.99) than G1 (p = 0.76) and G3 (p = 0.82). In G2, 53% (8/15) of the dogs exhibited increased antibody titers, whereas, in G3 and G1, 40% (6/15) and 27% (4/15) of the dogs exhibited increased antibody titers, respectively.

**Table 3:** Mean and standard deviation (95%CI) of the parasite loads analyzed by qPCR (copies/µL) in the three groups of treated dogs. P values ​​were obtained with the paired t-test.

**Miltefosine (n = 15)**		
**D0**	**D29**	**P-value**
4.5x10^7^ ± 1.5x10^8^	4.6x10^6^ ± 1.2x10^7^	0.05
**Allopurinol (n = 15)**		
**D0**	**D29**	**P-value**
1.2x10^7^ ± 2.3x10^7^	1.1x10^6^ ± 2.8x10^6^	< 0.01
**Miltefosine + Allopurinol (n = 15)**		
**D0**	**D29**	**P-value**
1.2x10^7^ ± 1.9x10^7^	1.0x10^6^ ± 1.3x10^6^	< 0.01

The values described are the mean ± SD (95%CI) of the amount of DNA in the skin of dogs on D0 and D29 of the groups treated with three different protocols. P values were obtained with paired t-test.

### qPCR analysis of parasite load

*Leishmania infantum* DNA was detected by qPCR in 100% of the skin samples on D0 and on D29. After 28 days of therapy, the qPCR analysis indicated a decrease in the mean parasite load from the three treatment groups (Table 3). However, changes recorded between the two time periods were statistically significant only when quantifying the skin biopsies in dogs treated with allopurinol and with the combination of both drugs (p < 0.01), the data shown in Table 3.

### Correlation between variables

The parasite load in all three treatment groups showed moderate positive correlation with the clinical score and showed no correlation with IFAT titer (Table 4).

**Table 4:** Correlation (r) found between the variables *Leishmania infantum* parasite load by qPCR in skin with clinical signs and parasite load with antibody titers by IFAT technique.

	**qPCR X Clinical Signs**	**qPCR X IFAT**
	(r)	(r)
**Miltefosine**	0.60	0.17
**Allopurinol**	0.55	0.00
**Milefosine + Allopurinol**	0.60	0.04

## Discussion

Miltefosine and allopurinol have been used in several studies as therapies for CVL, with varying results (Manna et al., 2008; Miró et al., 2009, 2011; Woerly et al., 2009; Nogueira et al., 2019; Iarussi et al., 2020). In clinical practice, the primary goal of CVL treatment is to improve the quality of life of the animal, as no cure is available. We did not observe a clinical cure in any of the dogs in the groups. However, the three groups of dogs responded successfully to the treatment regimens described in this study, demonstrating evident clinical improvement and a significantly reduced parasite load in the skin at the end of the 28-day treatment period.

Changes in the skin, ocular changes, lymphadenomegaly, anorexia, and apathy are frequently described in CVL studies; although these symptoms are common clinical signs of other diseases, they should be carefully investigated in dogs from endemic regions (Ciaramella et al., 1997; Amusategui et al., 2003; Manna et al., 2009). Mato Grosso, the Central-West region of Brazil, is endemic for VL, with the prevalence in canines ranging from 4.2% (Dias et al., 2017) to 35.6% (Brito et al., 2014).

Although this study evaluated the efficacy over a short period, miltefosine and allopurinol monotherapy, and combination therapy yielded evident clinical improvement. However, the percentage of clinical score reduction on D29 was more significant in the allopurinol group (G2). This result may be explained using a higher allopurinol dose than the therapeutic range (10–30 mg/kg/day) previously reported for CVL (Ginel et al., 1998, Manna et al., 2008; Nascimento et al., 2020). Hematological, biochemical and urinalysis tests were used for monitoring during and after treatments and no animals manifested xanthinuria or any other side effects using this dosage for 28 days.

Allopurinol can be safely used for long periods because of its low toxicity and cost. Leishmaniostatic action yields clinical improvement, reduces the parasite load, and prevents recurrence (Noli & Auxilia, 2005; Torres et al., 2016). Nascimento et al. (2020) reported that allopurinol monotherapy promoted a sharp drop in clinical scores during the first 60 days of treatment. However, allopurinol combined with immunotherapy generated a prolonged elimination of the parasite, strongly suggesting that this protocol could potentially inhibit vector transmission. Vercammen & De Deken (1996) and Lester & Kenyon (1996) reported satisfactory clinical improvement in dogs after 1–3 months of treatment. Koutinas et al. (2001) and Vercammen et al. (2002) demonstrated that allopurinol gradually reduced clinical signs.

Miltefosine as a monotherapy and in combination with allopurinol produced promising results and therefore should be considered an effective treatment, although the high cost is a disadvantage. Nogueira et al. (2019) reported a significant reduction in clinical scores after administering miltefosine at a dose of 2 mg/kg for four weeks. Woerly et al. (2009) reported reduced clinical scores in 43.5% and 61.2% of dogs after 28 and 56 days of treatment, respectively. Andrade et al. (2011) reported progressive clinical improvement and complete remission in 50% of dogs after treatment and over the subsequent 24 months. The explanation for this finding is the long half-life (approximately 159 h) in dogs, which characterizes the pharmacokinetics of the drug and results in a large drug accumulation in the body during treatment (Sindermann & Engel, 2006; Dorlo et al., 2012).

Antibody titer was not a relevant parameter for monitoring treatment in this study, as the protocols did not reduce antibody levels in 28 days. The reduction of antibody titers to non-significant levels usually occurs over time with continued allopurinol therapy (Miró et al., 2009; Santos et al., 2019; Iarussi et al., 2020). One of the criteria for long-term treatment discontinuation is the reversal to a negative antibody titer or a significant decrease in the initial titer (Kasabalis et al., 2020). The short time interval may not have influenced the serum antibody titers as much as it influenced the parasite load in the skin. The skin is the site of the first interaction between *Leishmania* and the host's immune system, and parasite detection in the skin samples is possible even in clinically healthy patients (Ciaramella et al., 1997). According to Scorza et al. (2021), the amount of parasite DNA in the skin is more relevant than the dog’s clinical status.

We observed a moderate positive correlation between the amount of parasite DNA per microliter and clinical score. Chagas et al. (2021) stated that the skin is affected regardless of the clinical score, although they identified a positive correlation between the skin parasite load and the clinical score. When we analyzed the statistical data representing the influence of the three protocols on the quantity of detected DNA, the data from the allopurinol monotherapy group and the association with miltefosine was noteworthy because of the promising results in the skin. The dogs treated with allopurinol, either alone or in combination with miltefosine, received 40 mg/kg/day of the drug, which is higher than the dose typically used in short- and long-term protocols (10–30 mg/kg/day) (Ginel et al., 1998, Manna et al., 2008; Nascimento et al., 2020).

Treatment with miltefosine and allopurinol was discontinued at 28 days. However, the animals continued to receive supportive pharmacological treatment after this period, for ethical reasons. Subsequent evaluations were not included in this study so that these additional drugs would not interfere with the results already achieved with these two drugs. Miltefosine monotherapy also provided satisfactory results, significantly reducing the parasite load in the skin. Nogueira et al. (2019) observed a greater reduction in the amount of parasite DNA in the skin 6–12 weeks after treatment. This indicates that, over time, the treatment effects can become more significant due to the prolonged activity of miltefosine. The combination of these drugs is the protocol recommended by several studies since the synergistic effect of the drugs, one leishmanicidal and the other leishmaniostatic, improves clinical symptoms and reduces the parasite load in several tissues over long periods (Manna et al., 2009, 2015; Dias et al., 2020; Iarussi et al., 2020). However, the prognosis varies according to the clinical status of the dog and the immune response to the treatment (Dantas-Torres et al., 2012).

In this study, we demonstrated that, even in the short term, it is possible to detect the effects of pharmacotherapy for the treatment of CVL with the only two oral drugs currently available on the market, miltefosine and allopurinol. However, our findings also reinforced the idea that short-term treatment monitoring using serology as a parameter is not possible. And qPCR is essential for monitoring the parasite load in infected tissues and very useful in a short-term assessment. As dogs are considered the main urban reservoirs of this disease, reducing the parasite load, mainly on the skin, reduces their ability to transmit *L. infantum* to the sandfly vector, consequently reducing the number of human infections (Ribeiro et al., 2018; Nogueira et al., 2019; Chagas et al., 2021).

## Conclusion

Miltefosine and allopurinol, alone or in association, promoted clinical improvement and reduced the burden of *L. infantum* in skin in dogs with canine visceral leishmaniasis in just 28 days of treatment. Skin qPCR exhibited reliable performance for DNA detection and quantification even at low concentrations. These reasons demonstrate that the skin is the ideal sample for the diagnosis and monitoring of *L. infantum* infection.

## References

[B001] Amusategui I, Sainz A, Rodríguez F, Tesouro MA (2003). Distribution and relationships between clinical and biopathological parameters in canine leishmaniasis. Eur J Epidemiol.

[B002] Andrade HM, Toledo VPCP, Pinheiro MB, Guimarães TMPD, Oliveira NC, Castro JA (2011). Evaluation of miltefosine for the treatment of dogs naturally infected with *L. infantum* (= *L. chagasi*) in Brazil. Vet Parasitol.

[B003] Borges LM, Oliveira AG, Mateus NLF, Oliveira EF, Arrua AEC, Infran JOM (2021). Canine Visceral Leishmaniasis in an Area of Sporadic Transmission in Brazil. Vector Borne Zoonotic Dis.

[B004] Brasil (2014). Manual de Vigilância e Controle de Leishmaniose Visceral.

[B005] Brasil (2019). Guia de vigilância em Saúde.

[B006] Brito VN, Oliveira CM, Lazari P, Sousa VR (2014). Epidemiological aspects of visceral leishmaniasis in Jaciara, Mato Grosso, Brazil, 2003 to 2012. Rev Bras Parasitol Vet.

[B007] Chagas ÚMR, de Avelar DM, Marcelino AP, Paz GF, Gontijo CMF (2021). Correlations between tissue parasite load and common clinical signs in dogs naturally infected by *Leishmania infantum.*. Vet Parasitol.

[B008] Ciaramella P, Oliva G, Luna RD, Gradoni L, Ambrosio R, Cortese L (1997). A retrospective clinical study of canine leishmaniasis in 150 dogs naturally infected by *Leishmania infantum.*. Vet Rec.

[B009] Dahl EH, Hamdan HM, Mabrouk L, Matendechero SH, Mengistie TB, Elhag MS (2021). Control of visceral leishmaniasis in East Africa: fragile progress, new threats. BMJ Glob Health.

[B010] Dantas-Torres F, Solano-Gallego L, Baneth G, Ribeiro VM, de Paiva-Cavalcanti M, Otranto D (2012). Canine leishmaniosis in the Old and New Worlds: unveiled similarities and differences. Trends Parasitol.

[B011] Dias AFLR, Almeida ABPF, Cruz FACS, Silva RR, Rodrigues JY, Otsubo AAF (2017). Seroprevalence and spatial analysis of canine visceral leishmaniasis in the Pantanal region, Mato Grosso State, Brazil. J Zoonotic Dis Public Health.

[B012] Dias ÁFLR, Ayres EDCBS, de Oliveira Martins DT, Maruyama FH, de Oliveira RG, de Carvalho MR (2020). Comparative study of the use of miltefosine, miltefosine plus allopurinol, and allopurinol in dogs with visceral leishmaniasis. Exp Parasitol.

[B013] Dorlo TP, Balasegaram M, Beijnen JH, de Vries PJ (2012). Miltefosine: a review of its pharmacology and therapeutic efficacy in the treatment of leishmaniasis. J Antimicrob Chemother.

[B014] Francino O, Altet L, Sánchez-Robert E, Rodriguez A, Solano-Gallego L, Alberola J (2006). Advantages of real-time PCR assay for diagnosis and monitoring of canine leishmaniosis. Vet Parasitol.

[B015] Ginel PJ, Lucena R, López R, Molleda JM (1998). Use of allopurinol for maintenance of remission in dogs with leishmaniasis. J Small Anim Pract.

[B016] Hong A, Zampieri RA, Shaw JJ, Floeter-Winter LM, Laranjeira-Silva MF (2020). One health approach to leishmaniases: understanding the disease dynamics through diagnostic tools. Pathogens.

[B017] Iarussi F, Paradies P, Foglia Manzillo V, Gizzarelli M, Caratozzolo MF, Navarro C (2020). Comparison of two dosing regimens of miltefosine, both in combination with allopurinol, on clinical and parasitological findings of dogs with leishmaniosis: a pilot study. Front Vet Sci.

[B018] Kasabalis D, Chatzis MK, Apostolidis K, Petanides T, Athanasiou LV, Xenoulis PG (2020). A randomized, blinded, controlled clinical trial comparing the efficacy of aminosidine (paromomycin)-allopurinol combination with the efficacy of meglumine antimoniate-allopurinol combination for the treatment of canine leishmaniosis due to *Leishmania infantum.*. Exp Parasitol.

[B019] Koutinas AF, Saridomichelakis MN, Mylonakis ME, Leontides L, Polizopoulou Z, Billinis D (2001). A randomised, blinded, placebo-controlled clinical trial with allopurinol in canine leishmaniosis. Vet Parasitol.

[B020] Lachaud L, Marchergui-Hammami S, Chabbert E, Dereure J, Dedet J, Bastien P (2002). Comparison of six PCR methods using peripheral blood for detection on canine visceral leishmaniasis. J Clin Microbiol.

[B021] Lester SJ, Kenyon JE (1996). Use of allopurinol to treat visceral leishmaniosis in a dog. J Am Vet Med Assoc.

[B022] Manna L, Corso R, Galiero G, Cerrone A, Muzj P, Gravino AE (2015). Long-term follow-up of dogs with leishmaniosis treated with meglumine antimoniate plus allopurinol versus miltefosine plus allopurinol. Parasit Vectors.

[B023] Manna L, Gravino AE, Picillo E, Decaro N, Buonavoglia C (2008). *Leishmania* DNA quantification by Real-time PCR in naturally infected dogs treated with Miltefosine. Ann N Y Acad Sci.

[B024] Manna L, Reale S, Vitale F, Gravino AE (2009). Evidence for a relationship between *Leishmania* load and clinical manifestations. Res Vet Sci.

[B025] Marcondes M, Day MJ (2019). Current status and management of canine leishmaniasis in Latin America. Res Vet Sci.

[B026] Miró G, Gálvez R, Fraile C, Descalzo MA, Molina R (2011). Infectivity to *Phlebotomus perniciosus* of dogs naturally parasitized with *Leishmania infantum* after different treatments. Parasit Vectors.

[B027] Miró G, Oliva G, Cruz I, Cañavate C, Mortarino M, Vischer C (2009). Multicentric, controlled clinical study to evaluate effectiveness and safety of miltefosine and allopurinol for canine leishmaniosis. Vet Dermatol.

[B028] Nascimento LFM, Miranda DFH, Moura LD, Pinho FA, Werneck GL, Khouri R (2020). Allopurinol therapy provides long term clinical improvement, but additional immunotherapy is required for sustained parasite clearance, in *L. infantum*-infected dogs. Vaccine: X.

[B029] Nogueira FS, Avino VC, Galvis-Ovallos F, Pereira-Chioccola VL, Moreira MAB, Romariz APPL (2019). Use of miltefosine to treat canine visceral leishmaniasis caused by *Leishmania infantum* in Brazil. Parasit Vectors.

[B030] Noli C, Auxilia ST (2005). Treatment of canine old world visceral leishmaniasis: a systematic review. Vet Dermatol.

[B031] Nunes JB, Coura-Vital W, Colombo FA, Baêta FJM, Pinheiro AC, Roatt BM (2018). Comparative analysis of real-time PCR assays in the detection of canine visceral leishmaniasis. Parasitol Res.

[B032] Pennisi MG, Reale S, Giudice SL, Masucci M, Caracappa S, Vitale M (2005). Real-time PCR in dogs treated for leishmaniasis with allopurinol. Vet Res Commun.

[B033] Proverbio D, Spada E, Bagnagatti De Giorgi G, Perego R (2014). Failure of miltefosine treatment in two dogs with natural *Leishmania infantum* infection. Case Rep Vet Med.

[B034] Quaresma PF, Murta SM, Ferreira EC, da Rocha-Lima AC, Xavier AA, Gontijo CM (2009). Molecular diagnosis of canine visceral leishmaniasis: identification of *Leishmania* species by PCR-RFLP and quantification of parasite DNA by real-time PCR. Acta Trop.

[B035] R Core Team (2019). R: A language and environment for statistical computing.

[B036] Ribeiro RR, Michalick MSM, da Silva ME, Dos Santos CCP, Frézard FJG, da Silva SM (2018). Canine Leishmaniasis: an overview of the current status and strategies for control. BioMed Res Int.

[B037] Sambrook J, Russel DW (2001). Molecular cloning: a laboratory manual.

[B038] Santos CCP, Ramos GS, De Paula RC, Faria KF, Moreira POL, Pereira RA (2020). Therapeutic efficacy of a mixed formulation of conventional and PEGylated Liposomes containing meglumine antimoniate, combined with allopurinol, in dogs naturally infected with *Leishmania infantum.*. Antimicrob Agents Chemother.

[B039] Santos CVB, Sevá AP, Werneck GL, Struchiner CJ (2021). Does deforestation drive visceral leishmaniasis transmission? A causal analysis. Proc Royal Soc B.

[B040] Santos MF, Alexandre-Pires G, Pereira MA, Marques CS, Gomes J, Correia J (2019). Meglumine antimoniate and miltefosine combined with allopurinol sustain pro-inflammatory immune environments during canine leishmaniosis treatment. Front Vet Sci.

[B041] Scorza BM, Mahachi KG, Cox AC, Toepp AJ, Leal-Lima A, Kumar Kushwaha A (2021). *Leishmania infantum* xenodiagnosis from vertically infected dogs reveals significant skin tropism. PLoS Negl Trop Dis.

[B042] Sevá ADP, Ferreira F, Amaku M (2020). How much does it cost to prevent and control visceral leishmaniasis in Brazil? Comparing different measures in dogs. PLoS One.

[B043] Sindermann H, Engel J (2006). Development of miltefosine as an oral treatment for leishmaniasis. Trans R Soc Trop Med Hyg.

[B044] Solano-Gallego L, Koutinas A, Miró G, Cardoso L, Pennisi MG, Ferrer L (2009). Directions for the diagnosis, clinical staging, treatment, and prevention of canine leishmaniosis. Vet Parasitol.

[B045] Torres M, Pastor J, Roura X, Tabar MD, Espada Y, Font A (2016). Adverse urinary effects of allopurinol in dogs with leishmaniasis. J Small Anim Pract.

[B046] Vercammen F, De Deken R (1996). Antibody kinetics during allopurinol treatment in canine leishmaniasis. Vet Rec.

[B047] Vercammen F, Fernandez-Perez FJ, del Amo C, Alunda JM (2002). Follow-up of *Leishmania infantum* naturally infected dogs treated with allopurinol: immunofluorescence antibody test, ELISA and Western blot. Acta Trop.

[B048] Woerly V, Maynard L, Sanquer A, Eun HM (2009). Clinical efficacy, and tolerance of miltefosine in the treatment of canine leishmaniosis. Parasitol Res.

[B049] WHO (2021). Leishmaniasis.

[B050] Yasur-Landau D, Jaffe CL, Doron-Faigenboim A, David L, Baneth G (2017). Induction of allopurinol resistance in *Leishmania infantum* isolated from dogs. PLoS Negl Trop Dis.

